# An insight into the botanical origins of propolis from permanent preservation and reforestation areas of southern Brazil

**DOI:** 10.1038/s41598-021-01709-1

**Published:** 2021-11-11

**Authors:** Alan Giovanini de Oliveira Sartori, Fernanda Papa Spada, Victor Pena Ribeiro, Pedro Luiz Rosalen, Masaharu Ikegaki, Jairo Kenupp Bastos, Severino Matias de Alencar

**Affiliations:** 1 Department of Agri-Food Industry, Food and Nutrition, Luiz de Queiroz College of Agriculture, University of São Paulo, Piracicaba, SP CEP: 13418-900 Brazil; 2 Department of Food and Experimental Nutrition, Food Research Center, University of São Paulo, 580, São Paulo, SP CEP 05508-000 Brazil; 3 Laboratory of Pharmacognosy, School of Pharmaceutical Sciences of Ribeirão Preto, University of São Paulo, Ribeirão Preto, SP CEP: 14040-903 Brazil; 4 Federal University of Alfenas, Alfenas, MG CEP: 37130-001 Brazil; 5 Department of Biosciences, Piracicaba Dental School, University of Campinas, Piracicaba, SP CEP: 13414-903 Brazil; 6 Faculty of Pharmaceutical Sciences, Federal University of Alfenas, Alfenas, MG CEP: 37130-001 Brazil

**Keywords:** Chemical biology, Ecology, Ecology, Chemistry

## Abstract

Brown propolis from permanent preservation and reforestation areas of southern Brazil have attracted international commercial interest and have a unique composition, although little is known about their botanical origins, which are the plant resins used by bee foragers to produce propolis. Hence, the volatile profiles of organic and non-organic brown propolis and resins of suspected botanical origins—*Araucaria angustifolia*, *Pinus elliott* and *Pinus taeda*—were determined using static headspace gas chromatography coupled to mass spectrometry (SHS-GCMS) and compared. Nighty nine volatiles were tentatively identified, and monoterpenes and sesquiterpenes were the most abundant classes. Principal component analysis (PCA) showed similarity between organic propolis and *A. angustifolia* volatile profiles (p < 0.05). Hierarchical clustering analysis showed singularities among propolis, even between propolis produced 1 km away from each other. Heatmaps were used to identify peaks present in similar relative intensities in both propolis and conifer resins. Hence, the approach using volatile profiles shed light to propolis botanical origins, which is important for authentication and traceability purposes.

## Introduction

Bees produce propolis, also known as bee glue, to strengthen and cover holes and cracks in the beehive walls, protect the hives against wind and water, reduce microbial growth and keep the temperature inside it^1,2^. Propolis is composed mainly of wax and plant resins and has long been used in ancient medicine due to its health effects, as well as food ingredient more recently. Bee foragers obtain the resins from vegetative buds and plant exudates near the beehives, and consequently, the chemical composition and the biological activities of propolis vary according to the region and the availability of botanical species where it is from Ref.^2^. The volatile compounds present in propolis contribute to its characteristic aroma and are biologically active substances from the resins, corresponding to up to 3% of propolis composition^1^.

The most common propolis worldwide are from temperate zones, where poplar trees (*Populus* sp.) are widespread and used as significant resin sources by bees to produce them. Propolis from temperate zones has high flavonoid aglycones and esters of substituted cinnamic acids as major non-volatile constituents. Sesquiterpenes aromatic compounds are major volatile constituents^1^. However, propolis produced particularly in Brazilian subtropical zones, is poor in flavonoids and contains monoterpenes as major volatiles^3,4^. Some of that propolis are certified as organically produced in permanent preservation areas and reforestation areas in Paraná and Santa Catarina states and have attracted attention due to their mild flavor and absence of heavy metals and pesticides. Moreover, they have shown relevant antioxidant, antimicrobial and anti-inflammatory activities, being the organic propolis variant 1 (OP1) the most widely organic propolis produced in that region^4^. Therefore, the investigation of the diverse botanical origin based on the chemical composition of OP1 is essential for authentication and traceability purposes, to complement studies concerning its biological activity, and to boost its potential use in the food industry.

*Araucaria angustifolia,* also known as Paraná pine or Brazilian pine (Araucariaceae family), is a critically endangered extinction species of subtropical to temperate rainforests in southern Brazil adjacent areas and was suggested as one botanical origin of brown propolis from southern Brazil^5^. Nevertheless, non-native conifers, such as *Pinus* sp*.* (Pinaceae family), which also produce resins, have been planted in reforestation areas near permanent preservation areas with natural forests, and apiary workers have witnessed Africanized *Apis mellifera* bees foraging them. Conifers have been reported as secondary botanical origins of propolis produced in temperate zones^6^. The approach of observing bees foraging behaviors and preferences have been helpful to identify the botanical origins of different types of propolis^7^, including the Brazilian red propolis^8^.

The botanical origins of propolis are commonly investigated by looking for few chemical markers or by overlapping chromatogram fingerprints without identifying the metabolites. In this context, an omics approach, which considers the extraction of the more volatile metabolites as possible with further data treatment using multivariate statistical analysis, was used for the first time in this study to shed light on the botanical origins of organic and non-organic brown propolis produced in permanent preservation areas and reforestation areas of southern Brazil.

## Methods

### Samples

Seven propolis samples were collected in apiaries located in two municipalities of Paraná state where OP1 is produced (Table 1). Five samples were from hives that hold organic certification from national (IMO Control, processes n° PR106 and 12-0030) and international organizations (the National Organic Program of the United States Department of Agriculture, processes n° 22422 and 23511, and Kiwa BCS Öko-Garantie GmbH 2016, processes n° A-2016-00005/2016-01341, A-2016-00005/2016-01342, 22422 and 23511). Battens were put in place in the beehives a few days before propolis collection (Table 1), which was carried out based on standard procedures^9^.

**Table 1:** Propolis and resin samples from permanent preservation areas and reforestation areas of southern Brazil.

ID	Municipality	Apiary	Latitude	Longitude	Altitude (m)	Date of batten placement (propolis) or date of wound generation (resins)	Date of propolis or resin collection	Type of propolis
**Propolis**								
P1	General Carneiro	‘Dois tanques’	25° 33′ 53, 27″	51° 19′ 38, 21″	1001	22/11/2019	10/12/2019	Organic (OP1)
P2	General Carneiro	‘Beira do mato’	26° 31′ 23, 36″	51° 16′ 39, 56″	1045	22/11/2019	10/12/2019	Organic (OP1)
P3	General Carneiro	‘Beira do mato’	26° 31′ 23, 36″	51° 16′ 39, 56″	1045	24/11/2019	10/12/2019	Organic (OP1)
P4	União da Vitória	‘Vila Zulmira sede’	26° 19′ 43, 63″	51° 11′ 35, 62″	867	24/11/2019	10/12/2019	Non-organic
P5	União da Vitória	‘Vila Zulmira sede’	26° 19′ 43, 63″	51° 11′ 35, 62″	867	24/11/2019	09/12/2019	Non-organic
P6	União da Vitória	‘Vila Zulmira serra’	26° 11′ 12, 65″	51° 07′ 17, 82″	867	24/11/2019	10/12/2019	Organic (OP1)
P7	União da Vitória	‘Vila Zulmira serra’	26° 11′ 12, 65″	51° 07′ 17, 82″	867	24/11/2019	10/12/2019	Organic (OP1)
**Resins**								
AA1	União da Vitória	–	26° 11′ 12, 65″	51° 07′ 17, 82″	867	07/12/2019	09/12/2019	–
AA2	União da Vitória	–	26° 11′ 12, 65″	51° 07′ 17, 82″	867	07/12/2019	09/12/2019	–
PT	General Carneiro	–	26° 31′ 23, 36″	51° 16′ 39, 56″	1045	08/12/2019	10/12/2019	–
PE1	General Carneiro	–	26° 31′ 23, 36″	51° 16′ 39, 56″	1045	08/12/2019	10/12/2019	–
PE2	União da Vitória	–	26° 11′ 12, 65″	51° 07′ 17, 82″	867	07/12/2019	09/12/2019	–

*OP1* organic propolis variant 1^4^.

Resin samples were collected from wounds generated two days before collection, according to standard procedures^9^, on December 9th and 10th of 2019 from *Pinus elliott* (PE), *Pinus taeda* (PT) and *Araucaria angustifolia* (AA) individuals located near the beehives (Table 1). Only one individual of PT produced resin by the date of collection, while it was possible to collect resins of two individuals of PE (PE1 and PE2) and AA (AA1 and AA2).

The botanical sources were defined based on literature^5,6^ and on reports of apiary workers who have seen bees collecting resins from them near the hives. Bees behavior and their plant resin sources preferences were monitored, photographically registered, and a video of bees collecting resin from a wound generated in one AA individual was recorded (Supplementary Video). It was not possible to film bees collecting resins from *Pinus* sp, since they were seen only in wounds on the top of those trees.

Botanical identification was possible with the help of a local biologist, who visually evaluated specific characteristics of AA^10^ and of each *Pinus* species (cones with peduncles and no thorns, abundant resin exudation in the stem wounds, and denser, longer and darker aciculas for PE, when compared with PT)^11^, and based on voucher specimens deposited in the herbarium of the College of Agriculture Luiz de Queiroz (University of São Paulo) with reference numbers ESA 105667 (*Araucaria angustifolia*), ESA 082760 (*Pinus taeda*) and ESA 000111 (*Pinus elliottii*).

All collections were conducted in private lands after permission was obtained from the land owners. Plant experiments were performed in accordance with national/institutional guidelines and regulations, and permission to study the chemical composition of OP1 and plant resins was obtained from the National System for the Management of Genetic Heritage and Associated Traditional Knowledge (SisGen) under the registration n° A5A0509.

### Untargeted analysis of volatile compounds

Samples of crude propolis (200 ± 2 mg) or crude resins (20 ± 0.2 mg) added with 1 µL internal control (l-carvone, Sigma-Aldrich, St. Louis, MO, USA) were placed in 20 mL glass vials and capped with gas-tight magnetic screw caps with a polytetrafluoroethylene/silicone septum for needle pierce. l-carvone was chosen as internal standard to normalize data for the multivariate analysis since it is a monoterpene not expected to be naturally present in this type of propolis.

For static headspace (SHS) extraction, crude propolis was incubated at 180 °C for 15 min in an AOC-5000 auto-injector (Shimadzu, Tokyo, Japan). Extraction conditions were defined based on the number and intensity of peaks in the total ion chromatogram (TIC), using temperature range of 40–180 °C at 10–15 min (Supplementary Fig. S1).

Then, samples were automatically injected into a GC-2010 coupled with a QP 2010 Plus mass spectrometer, using the GCMS solution (version 4.20) software (Shimadzu, Tokyo, Japan). Desorption time was 3 min, and volatiles was separated in an Rtx-5MS column (30 m × 0.25 mm × 0.25 µm, Restek, Bellefonte, PA, USA). Injection temperature and ion source temperature was set at 200 °C, oven temperature started at 50 °C and was kept for 3 min, increased at 120 °C at a 5 °C/min rate, and then increased up to 250 °C at a 10 °C/min rate. The carrier gas was helium at a flow rate of 1.0 mL/min. The mass spectrometer ionization energy was70 eV, and the spectrum was recorded from 45 *m/z* to 450 *m/z*, with a split mode of 30.

Retention time (RT) and mass-to-charge ratio (*m/z*) of duplicates were obtained using Mass Hunter software (Agilent, Santa Clara, CA, USA), with the deconvolution feature. Volatiles were tentatively identified by similarity with the libraries NIST (version 11), Wiley FFNSC Library (version 1.3) and Wiley (version 8) followed by calculation of linear retention index (LRI) using a series of saturated alkanes C7–C30 (Supelco, Bellefonte, PA, EUA), and then confirmation by comparing the calculated LRI with literature data. Volatiles were semiquantified by relative intensity (%) in the TIC. Some unidentified terpenes displayed ions of *m/z* typical of terpenes (136 or 138) in their mass spectra^12^.

### Multivariate analysis

The data generated (peak list with RT and intensities in the TIC and tentative identification) were uploaded online MetaboAnalyst software, v. 5.0 (https://www.metaboanalyst.ca/) to carry out multivariate statistical analysis. Data were normalized by an internal control (the terpene l-carvone), using autoscaling (mean-centered and divided by the standard deviation of each variable), and non-hierarchical principal component analysis (PCA), and hierarchical clustering heatmap were performed. The level of confidence was 95%.

## Results

Samples are described at Table 1. The main classes of volatiles were monoterpene and sesquiterpene for all samples (Table 2). Monoterpenes corresponded to most resins' TIC of PT and PE, and more than 86% of the TIC of P2 and P3. Relevant intraspecies variation was observed for AA, with monoterpenes proportion varying from 78 to 89%, while there was no relevant variation between the two PE individuals (98.5% and 98.3%). It is important to mention that the period between wounds generation and resins collection were the same for all samples (Table 1), and that they were stored in equal flasks during the same period until analysis, that was carried out under the same conditions. Therefore, variables other than collection and analytical methodologies may explain the variation that were not investigated in our study.

**Table 2:** Classes of volatiles obtained by SHS-GCMS in brown propolis and conifer resins from southern Brazil.

Class	Samples (% TIC)											
	P1	P2	P3	P4	P5	P6	P7	AA1	AA2	PT	PE1	PE2
**SHS-GCMS**												
Acid	–	–	–	0.2	0.3	1.4	0.6	–	–	–	–	–
Alcohol	–	–	–	–	1.1	7.9	6.7	–	–	–	–	–
Aldehyde	3.6	0.8	1.6	2.1	10.1	2.2	1.3	–	–	2.0	0.7	0.7
Ester	0.0	0.0	0.0	1.1	1.7	–	0.5	0.0	2.7	0.0	–	–
Ether	0.0	–	–	–	0.7	2.5	1.3	–	–	–	–	–
Hydrocarbon	–	–	–	–	0.9	0.8	0.4	–	–	–	–	–
Ketone	–	–	–	–	0.3	0.6	0.3	–	–	–	–	–
Monoterpene	68.3	89.8	86.6	68.1	51.2	34.4	23.1	89.3	78.2	94.7	98.5	98.3
Sesquiterpene	16.7	1.8	6.8	23.7	11.3	23.1	47.4	9.3	19.1	1.4	–	–
Unidentified terpene	–	–	–	–	0.4	1.4	2.1	–	–	–	–	–
Non-identified	11.4	7.6	5.0	4.8	22.0	25.7	16.3	1.4	–	1.9	0.8	1.0

– : Not detected or < 0.00. P1: Propolis from the ‘Dois tanques’ apiary in General Carneiro municipality, P2 and P3: Propolis from the ‘Beira do mato’ apiary in General Carneiro municipality. P4 and P5: Propolis from the ‘Vila Zulmira sede’ apiary in União da Vitória municipality. P6 and P7: Propolis from the ‘Vila Zulmira serra’ apiary in União da Vitória municipality. AA: *Araucaria angustifolia* resins. PT: *Pinus taeda* resins. PE: *Pinus elliott* resins.

In the propolis samples, the proportion of sesquiterpenes was more significant only for P7, which was very different from P6, although both samples were collected at the same apiary, but from different beehives. Other chemical classes found in the samples were acid, alcohol, aldehyde, ester, ether, furan, ketone and aromatic hydrocarbons.

Typical ions up to 2.07% indicated the presence of unidentified terpenes. The volatile profile diversity among propolis samples may indicate bees collect resins from diverse plant resins sources.

Supplementary Tables S1, S2 and S3 online show the tentatively identified volatiles extracted by SHS-GCMS, their LRI, and the percentage of the total peak area of the TIC. The number of detected peaks was 141, of which 99 were tentatively identified, and one unidentified terpene was found based on the presence of ions with typical *m/z* (*m/z* 136 or 138) in the mass spectra. Retention times, LRI, when calculated, and the percentage of the total peak area of the TIC of the non-identified compounds, including the unidentified terpene, can be found as Supplementary Table S4 online.

Alpha-pinene was the most abundant volatile found in all propolis, varying from 18.2% in P6 to 61.5% in PE1, except for P7. β-Pinene was the second most abundant volatile found in propolis samples from General Carneiro (P1, P2 and P3) and in the ‘Vila Zulmira sede’ apiary (P4 and P5), varying from 8.3% (P5) to 20.3% (P1), as well as in *Pinus* resins (PT, PE1 and PE2), varying from 16.1% (PE1) to 25.9% (PE2).

Furfural was notably found as the fourth most abundant volatile in P5 (4.9%), while present in proportions from 0.4 to 0.8% in P1, P2, P4 and P6. The most abundant volatiles in P7 were α-bisabolol (9.7%), α-pinene (9.5%), β-eudesmol (8.1%) and γ-eudesmol (6.1%). β-Eudesmol (6.7%) and γ-eudesmol (5.7%) were also relevant in P6.

**Figure 1:**
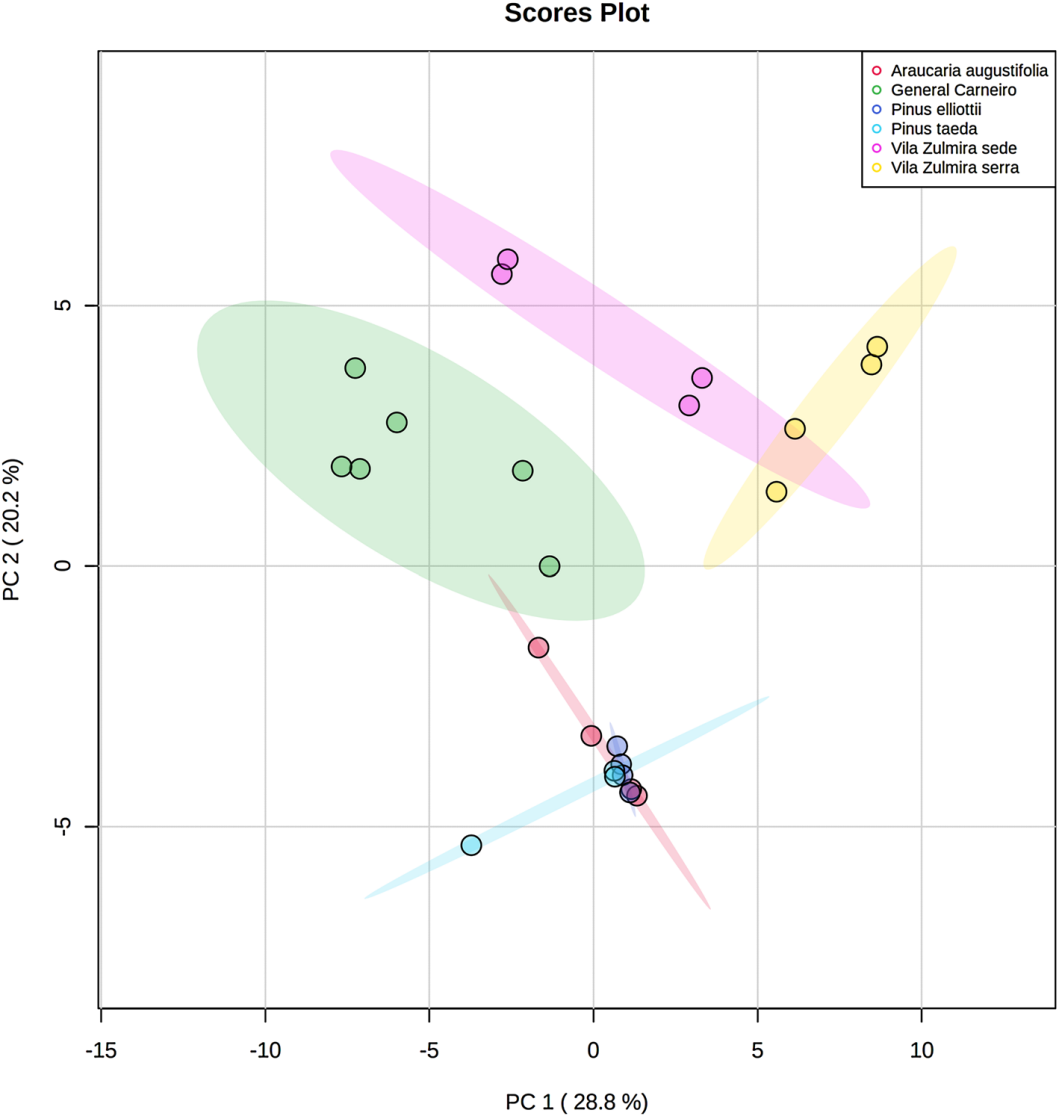
Pairwise score plots between the two principal components for chromatograms obtained by SHS-GCMS from brown propolis and conifer resins from southern Brazil. The explained variances are shown in the corresponding axes and the overlaps of the colored shaded areas indicate similarities among propolis from General Carneiro, ‘Vila Zulmira sede’ and ‘Vila Zulmira serra’ and resins of *Araucaria angustifolia*, *Pinus taeda* and *Pinus elliotti* at a level of confidence of 95%. Figure was generated by the MetaboAnalyst software, v. 5.0 (https://www.metaboanalyst.ca/).

All peak intensity data of the chromatograms were used for multivariate analysis (non-hierarchical PCA and hierarchical clustering heatmap) to investigate similarities among them. For that, samples were grouped. The apiaries from União da Vitória municipality were differentiated, since propolis from ‘Vila Zulmira sede’ apiary is non-organic and propolis from ‘Vila Zulmira serra’ is organic. Resins samples were grouped according to their species. Then, PCA was performed, considering volatiles found in proportions > 1% in the TIC (Fig. 1).

The sum of the two principal components totalized 49.0%, which means that the model was successful in explaining ca. 50% of the variances. As shown in Fig. 1, the chromatograms of organic propolis from General Carneiro municipality, represented by the green points within the green shaded area, overlapped with the AA group, meaning that they are similar at a level of confidence of 95%. Similarly, the *Pinus* sp. groups overlapped with the AA group, and thus, it was not possible to differentiate them at a family level (Fig. 1). Concerning propolis groups, the chromatograms from both apiaries of União da Vitória municipality (‘Vila Zulmira sede’ and ‘Vila Zulmira serra’) overlapped (p < 0.05), and both were spatially close to those from General Carneiro municipality (p > 0.05).

**Figure 2:**
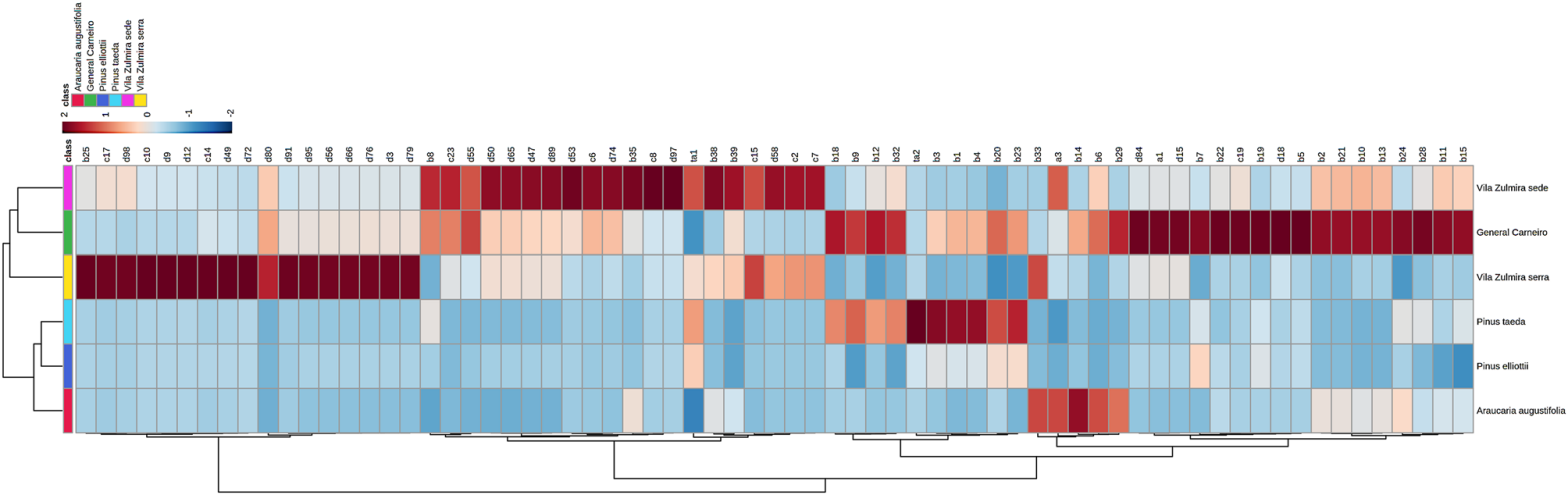
Hierarchical clustering heatmap of chromatograms obtained by SHS-GCMS from brown propolis and conifer resins from southern Brazil. Each colored cell on the heatmap indicates the correlation coefficient, and the scale code is shown on the top right corner (red and blue colors mean positive and negative correlations, respectively, at a level of confidence of 95%). General Carneiro, ‘Vila Zulmira sede’ and ‘Vila Zulmira serra’ refer to the origin of the propolis samples and *Araucaria angustifolia*, *Pinus taeda* and *Pinus elliotti* refer to the plant from where the resins were collected. Codes refer to volatiles shown in Supplementary Tables S1, S2 and S3. Figure was generated by the MetaboAnalyst software, v. 5.0 (https://www.metaboanalyst.ca/).

Hierarchical clustering in the form of heatmaps were then generated (Fig. 2), considering volatiles found in proportions > 1% in the TIC. In the dendrograms using Euclidian distance on the top of the columns, PE and PT were clustered together with the highest similarity. The resins (PE, PT and AA) were clustered together, while propolis was grouped in another cluster.

Additionally, the heatmaps illustrate that propolis from the distinct groups have singular chromatograms. It can be more easily seen in Fig. 2, where higher intensities from b25 to d79 are notable for ‘Vila Zulmira serra’ and from b8 to c7 for ‘Vila Zulmira sede’ and from b29 to b15 for General Carneiro. It is noteworthy that propolis from ‘Vila Zulmira sede’ is non-organic, while propolis from ‘Vila Zulmira serra’ is certified as organic, and those apiaries are located only 1 km away from each other.

The hierarchical clustering heatmaps were also used in our study to look for volatiles present in similar intensities in both propolis groups and in conifer resins groups aiming to find possible phytochemical markers. As shown at Fig. 2, b18 (α-campholenal), b9 (α-phellandrene), b32 (β-bourbonene) and b20 (*trans*-verbenol) were found in similar intensities in both General Carneiro and PT, as well as b29 (p-mentha-1,8-dien-7-ol) was found in both General Carneiro and AA.

## Discussion

Alpha-pinene, the most abundant volatile found in all propolis, was also the most abundant volatile found in propolis produced in the Adriatic Sea coast of Italy, and its likely botanical origin was suggested to be native conifer trees from that region^13^. α-Pinene together with β-pinene were previously found as the two most abundant volatiles in propolis produced in the Rio Grande do Sul^14^ and Paraná^3^ states, in a not specified Brazilian propolis sample^15^, and propolis from South Africa^16^ and Uruguay^1^. It is noteworthy that South Africa and Uruguay are in similar latitudes to southern Brazil, indicating a characteristic profile related to that propolis location.

Furfural, which was found in almost all samples, is a product of sugars dehydration commonly found in agricultural byproducts and was identified by SHS-GCxGC-TOF-MS in South African propolis, in which volatiles was extracted by heating at 45 °C/5 min, at concentrations ranging from trace to 11.3%^16^.

β-Eudesmol was found as the most abundant volatile in propolis produced in France, Hungary, Bulgaria, and Northern Italy and was also the most abundant volatile in the distilled essential oil of *Populus nigra* buds, which likely is its primary botanical origin^1^. However, in our study, β-eudesmol was also found in the resins of AA as a minor volatile (from 0.7 to 2.4%). Additionally, AA resins were the only ones containing sabinene, α-thujene and α-bisabolol. Thereby, AA may be plant sources of these volatiles for brown propolis from southern Brazil. α-Bisabolol was also found as a major volatile in propolis produced in temperate zones of China and Turkish^1^.

It is noteworthy that the temperature used to extract the volatiles, 180 °C, was higher than those commonly used for volatile profile characterization of 50–75 °C^3,15,17^. The degradation rate of pure monoterpenes at 120 °C varied greatly, depending on the compound, as it was 100% after 4 h for α-terpinene, 50% after 24 h for limonene, and 38% after 72 h for camphene^18^. The thermal degradation led to *p-*cymene, eucarvone and 1,2-epoxyde derivatives from limonene; thymol, ketoaldehydes, and eucalyptol from α-terpinene; and camphenilone, verbenone, and aromatic compounds from camphene^18^.

Although verbenone was found in samples of our study (up to 1.3%), it was also tentatively identified in brown propolis extracted at 75 °C/30 min^3^, while *p-*cymene and verbenone were tentatively identified in Mediterranean propolis extracted at 60 °C/45 min^17^.

Furthermore, McGraw et al.^18^ quoted Punsuvon, who reported the degradation at 90–130 °C (not specifying the time length) of pure α-pinene (23–37%), forming β-pinene, α-pinene oxide, α-campholenal, verbenol, pinocamphone, myrtenol and verbenone, and of pure β-pinene (22%), forming mainly myrtenol. From those thermal degradation products of α-pinene, some are reportedly relevant in propolis and conifer tissues, such as β-pinene, α-campholenal, myrtenol and verbenol^1,15,19^. Hence, the terpene diversity in natural products seems to result from naturally occurring chemical reactions catalyzed by microorganisms or enzyme systems^20^. At the same time, induced heating is a non-natural way to get it, and it is not simple to differentiate whether the terpene diversity is natural or induced by extraction conditions.

The increase in temperature increased peak intensities up to 180 °C, and the number of peaks also increased, which likely indicates volatiles release from the propolis's complex resinous/waxy matrix (Supplementary Fig. S1). However, the formation of low percentages of the tentatively identified carvone oxide (Supplementary Table S1), which likely had the added internal control l-carvone as a precursor, is an indication of oxidation. Nevertheless, l-carvone was pierced outside the samples within the vials. Thereby, it was more exposed to O_2_ and more prone to oxidation than the other volatiles present in the propolis/resins matrices.

Concerning the multivariate analysis, the PCA showed that the SHS-GCMS method was sensible to discriminate propolis samples produced in different municipalities, even when the distance between the apiaries was 72 km (from ‘Beira do mato’ to ‘Vila Zulmira sede’). Moreover, the PCA indicates that *A. angustifolia* may be more attractive than *Pinus* species for bee foragers as a plant resin source to produce propolis.

It is noteworthy that the possibility of *Araucaria* *sp.* resins be used as a botanical source for bees to produce brown propolis in southern Brazil was previously suggested by^5^, based on the identification of a single non-volatile compound, which is typical in some *Araucaria* species, in propolis samples from Paraná state. AA is a dominant species in subtropical and temperate rainforests in southern Brazil and adjacent areas. These areas were intensively explored over the nineteenth century. Nowadays, it is legally protected in permanent preservation areas since AA is endangered^10^. Therefore, the likely presence of AA resins in OP1 reinforces the need for sustainable preservation of natural environments since it may be related to OP1’s outstanding antioxidant activity^4^.

From the tentatively identified volatiles in the hierarchical clustering heatmap, α-campholenal, α-phellandrene, β-bourbonene and *trans*-verbenol were found in essential oils of *Pinus species*^19^*.* This finding may indicate PT as another plant resin source for propolis production in those areas. To our knowledge, *p*-mentha-1,8-dien-7-ol was tentatively identified in plants from the *Araucariceae* family for the first time. *p*-Mentha-1,8-dien-7-ol, also known as perilla alcohol, is found in many plants' essential oils, such as lavendin, peppermint, spearmint, and cherries^21^. Therefore, further studies should be conducted with authentic standards to confirm the identified volatiles in brown propolis and conifer resins from southern Brazil to be further used as phytochemical markers.

In conclusion, there are indications that the resin from native *Araucaria angustifolia* is more attractive for bees to produce propolis in southern Brazil, although there is also an indication that non-native *Pinus elliott* and *Pinus taeda* are plant resin sources as well. However, the singularities on the chromatograms of propolis from each apiary/municipality illustrated in the heatmaps and the not complete overlap of the propolis and the conifer resins in the PCA may indicate that there are other botanical sources for bees to produce propolis within the permanent preservation areas of southern Brazil, which remain unknown.

## Supplementary Information

Supplementary Information.

Supplementary Video 1.
