# Supplementary material for: An insight into the botanical origins of propolis from permanent preservation and reforestation areas of southern Brazil
Source: Sci Rep. 2021 Nov 11;11:22043. doi: 10.1038/s41598-021-01709-1 (PMC8586149; doi:10.1038/s41598-021-01709-1)
Supplement: Supplementary file 1 — Supplementary Information. [file 41598_2021_1709_MOESM1_ESM.docx]

**Supplementary information**

**An insight into the botanical origins of propolis from permanent preservation and reforestation areas of southern Brazil**

Alan Giovanini de Oliveira Sartori*, Fernanda Papa Spada, Victor Pena Ribeiro, Pedro Luiz Rosalen, Masaharu Ikegaki, Jairo Kenupp Bastos, Severino Matias de Alencar

*Corresponding author:

Alan Giovanini de Oliveira Sartori – Department of Agri-Food Industry, Food and Nutrition, Luiz de Queiroz College of Agriculture, University of São Paulo, CEP: 13418-900, Piracicaba, SP, Brazil. Orcid: 0000-0002-6782-5379; Phone: +55 19 34478686; Email: alangosartori@usp.br

Fernanda Papa Spada – Department of Food and Experimental Nutrition, Food Research Center, University of São Paulo, 580, CEP 05508-000, São Paulo, SP, Brazil.

Victor Pena Ribeiro – Laboratory of Pharmacognosy, School of Pharmaceutical Sciences of Ribeirão Preto, University of São Paulo, CEP: 14040-903, Ribeirão Preto, SP, Brazil.

Pedro Luiz Rosalen – Federal University of Alfenas, CEP: 37130-001, Alfenas, MG, Brazil; Department of Biosciences, Piracicaba Dental School, University of Campinas, CEP: 13414-903, Piracicaba, SP, Brazil.

Masaharu Ikegaki – Faculty of Pharmaceutical Sciences, Federal University of Alfenas, CEP: 37130-001, Alfenas, MG, Brazil.

Jairo Kenupp Bastos – Laboratory of Pharmacognosy, School of Pharmaceutical Sciences of Ribeirão Preto, University of São Paulo, CEP: 14040-903, Ribeirão Preto, SP, Brazil.

Severino Matias de Alencar – Department of Agri-Food Industry, Food and Nutrition, Luiz de Queiroz College of Agriculture, University of São Paulo, CEP: 13418-900, Piracicaba, SP, Brazil.

**Abstract**: Brown propolis from permanent preservation and reforestation areas of southern Brazil have attracted international commercial interest and have a unique composition, although little is known about their botanical origins, which are the plant resins used by bee foragers to produce propolis. Hence, the volatile profiles of organic and non-organic brown propolis and resins of suspected botanical origins – Araucaria angustifolia, Pinus elliott and Pinus taeda – were determined using static headspace gas chromatography coupled to mass spectrometry (SHS-GCMS) and compared. Nighty nine volatiles were tentatively identified, and monoterpenes and sesquiterpenes were the most abundant classes. Principal component analysis (PCA) showed similarity between organic propolis and A. angustifolia volatile profiles (p<0.05). Hierarchical clustering analysis showed singularities among propolis, even between propolis produced 1 km away from each other. Heatmaps were used to identify peaks present in similar relative intensities in both propolis and conifer resins. Hence, the approach using volatile profiles shed light to propolis botanical origins, which is important for authentication and traceability purposes.

*Keywords*: volatiles, Paraná pine, Araucaria, Pinus, bee, *Apis mellifera*

Figure S1. Overlap of SHS-GCMS chromatograms of a pooled sample made with all propolis samples and obtained after extraction using 40 °C/10 min (black), 100 °C/15 min (blue) and 180 °C/15 min (pink).

Table S1. Tentatively identified monoterpenes obtained by SHS-GCMS in brown propolis and conifer resins from southern Brazil.

| RT | Volatile | LRI | LRI Lit. | Code | Samples (% TIC) | | | | | | | | | | | |
| --- | --- | --- | --- | --- | --- | --- | --- | --- | --- | --- | --- | --- | --- | --- | --- | --- |
|  |  |  |  |  | P1 | P2 | P3 | P4 | P5 | P6 | P7 | AA1 | AA2 | PT | PE1 | PE2 |
| 8.660 | Tricyclene | 922 | 923^1^ | b1 | 0.7 | 2.3 | 1.6 | 0.8 | 1.3 | 0.0 | 0.5 | 0.5 | 0.7 | 2.8 | 2.2 | 2.8 |
| 8.818 | α-Thujene | 926 | 928^1^ | b2 | 2.2 | 2.7 | 5.1 | 4.7 | 1.2 | 0.3 | 0.2 | 5.2 | 2.4 | 0.0 | 0.0 | 0.0 |
| 9.085 | α-Pinene | 933 | 936^1^ | b3 | 21.7 | 34.5 | 29.2 | 21.7 | 21.0 | 18.2 | 9.5 | 32.0 | 35.2 | 54.3 | 61.5 | 52.5 |
| 9.500 | Camphene | 948 | 950^1^ | b4 | 2.2 | 3.6 | 2.7 | 1.4 | 1.8 | 0.9 | 1.8 | 1.9 | 1.8 | 4.6 | 4.6 | 4.5 |
| 9.672 | Thuja-2.4(10)-diene | 953 | 956^1^ | b5 | 2.2 | 4.4 | 3.5 | 1.3 | 2.1 | 2.7 | 0.8 | 1.6 | 2.0 | 2.1 | 1.3 | 2.3 |
| 10.305 | Sabinene | 973 | 973^1^ | b6 | 1.5 | 0.9 | 4.2 | 4.1 | 0.0 | 0.0 | 0.0 | 11.6 | 6.4 | 0.0 | 0.0 | 0.0 |
| 10.417 | β-Pinene | 976 | 978^1^ | b7 | 20.3 | 15.7 | 12.8 | 9.8 | 8.3 | 4.3 | 2.6 | 7.7 | 6.1 | 16.4 | 16.1 | 25.9 |
| 10.814 | Myrcene | 990 | 989^1^ | b8 | 1.8 | 0.7 | 1.8 | 4.5 | 1.5 | 0.8 | 0.7 | 0.6 | 0.6 | 2.4 | 1.5 | 0.3 |
| 11.259 | α-Phellandrene | 1004 | 1004^1^ | b9 | 0.9 | 1.3 | 1.2 | 0.6 | 1.4 | 1.2 | 0.5 | 1.3 | 0.9 | 0.9 | 0.0 | 0.0 |
| 11.645 | α-Terpinene | 1016 | 1017^1^ | b10 | 1.2 | 1.3 | 2.0 | 1.7 | 2.2 | 0.7 | 0.7 | 2.4 | 0.7 | 0.0 | 0.0 | 0.0 |
| 11.890 | p-Cymene | 1023 | 1024^1^ | b11 | 1.1 | 1.5 | 1.6 | 1.5 | 1.7 | 1.4 | 0.6 | 2.2 | 1.2 | 1.0 | 0.0 | 0.0 |
| 12.038 | Limonene | 1028 | 1030^1^ | b12 | 3.1 | 4.1 | 3.3 | 4.3 | 2.6 | 1.6 | 1.5 | 3.2 | 6.0 | 2.2 | 2.9 | 2.0 |
| 12.305 | *cis*-β-Ocimene | 1037 | 1038^1^ | c24 | 0.0 | 0.4 | 0.0 | 0.0 | 0.0 | 0.0 | 0.0 | 0.0 | 0.0 | 0.0 | 0.0 | 0.0 |
| 12.623 | *trans*-β-Ocimene | 1047 | 1048^1^ | c11 | 0.0 | 0.0 | 0.0 | 0.0 | 0.5 | 0.4 | 0.7 | 0.0 | 0.0 | 0.0 | 0.0 | 0.0 |
| 12.988 | γ-Terpinene | 1058 | 1060^1^ | b13 | 1.5 | 1.6 | 2.4 | 2.0 | 2.6 | 0.8 | 0.8 | 3.3 | 0.9 | 0.0 | 0.0 | 0.0 |
| 13.263 | *cis*-Sabinene hydrate | 1067 | 1067^1^ | b14 | 0.0 | 0.0 | 0.8 | 0.0 | 0.0 | 0.0 | 0.0 | 3.6 | 0.0 | 0.0 | 0.0 | 0.0 |
| 13.927 | Terpinolene | 1088 | 1087^1^ | b15 | 0.6 | 0.6 | 0.9 | 0.6 | 1.3 | 0.6 | 0.5 | 1.2 | 0.7 | 0.9 | 0.0 | 0.0 |
| 14.229 | Linalool | 1098 | 1099^1^ | b16 | 0.5 | 0.4 | 0.0 | 0.2 | 0.0 | 0.0 | 0.0 | 0.4 | 0.0 | 0.0 | 0.0 | 0.0 |
| 14.288 | Perillene | 1100 | 1099^1^ | a2 | 0.0 | 0.0 | 0.0 | 0.1 | 0.0 | 0.0 | 0.0 | 0.0 | 0.0 | 0.0 | 0.0 | 0.0 |
| 15.089 | α-Campholenal | 1126 | 1124^1^ | b18 | 0.6 | 1.8 | 1.3 | 0.3 | 0.4 | 0.1 | 0.0 | 0.7 | 1.1 | 0.8 | 0.6 | 0.7 |
| 15.514 | *trans*-Pinocarveol | 1140 | 1140^1^ | b19 | 1.3 | 3.2 | 2.5 | 0.7 | 0.0 | 0.0 | 0.0 | 0.9 | 1.0 | 1.6 | 1.2 | 2.3 |
| 15.679 | *trans*-Verbenol | 1145 | 1144^1^ | b20 | 0.0 | 1.0 | 1.4 | 0.5 | 0.0 | 0.0 | 0.0 | 1.2 | 2.1 | 0.9 | 1.5 | 1.8 |
| 16.027 | Sabina ketone | 1157 | 1156^1^ | d27 | 0.0 | 0.0 | 0.3 | 0.2 | 0.0 | 0.0 | 0.0 | 0.0 | 0.0 | 0.0 | 0.0 | 0.0 |
| 16.172 | *trans*-Pinocamphone | 1162 | 1162^1^ | d28 | 0.0 | 0.5 | 0.0 | 0.0 | 0.0 | 0.0 | 0.0 | 0.0 | 0.0 | 0.0 | 0.0 | 0.0 |
| 16.238 | Pinocarvone | 1164 | 1161^1^ | d29 | 0.6 | 0.4 | 0.5 | 0.0 | 0.0 | 0.0 | 0.0 | 0.0 | 0.0 | 0.0 | 0.0 | 0.0 |
| 16.285 | Borneol | 1166 | 1166^1^ | d19 | 0.0 | 0.0 | 0.0 | 0.0 | 0.6 | 0.0 | 0.0 | 0.0 | 0.0 | 0.0 | 0.0 | 0.0 |
| 16.326 | p-Mentha-1.5-dien-8-ol | 1167 | 1167^1^ | d30 | 0.0 | 0.9 | 0.8 | 0.6 | 0.0 | 0.0 | 0.9 | 0.0 | 0.0 | 0.0 | 0.0 | 0.0 |
| 16.664 | Terpinen-4-ol | 1177 | 1177^1^ | b21 | 0.4 | 0.9 | 1.3 | 1.5 | 0.0 | 0.0 | 0.0 | 1.8 | 0.0 | 0.0 | 0.0 | 0.0 |
| 16.914 | *trans*-1(7),8-p-Menthadien-2-ol | 1187 | 1187^1^ | d35 | 0.3 | 0.0 | 0.0 | 0.0 | 0.0 | 0.0 | 0.0 | 0.0 | 0.0 | 0.0 | 0.0 | 0.0 |
| 17.046 | α-Terpineol | 1190 | 1190^1^ | b22 | 0.5 | 1.4 | 1.0 | 0.7 | 0.3 | 0.2 | 0.3 | 0.8 | 0.0 | 0.0 | 0.1 | 0.7 |
| 17.236 | Myrtenol | 1197 | 1194^1^ | b23 | 1.3 | 2.1 | 2.2 | 1.7 | 0.4 | 0.4 | 0.2 | 1.6 | 1.4 | 2.3 | 4.9 | 1.9 |
| 17.606 | Verbenone | 1212 | 1206^1^ | b24 | 0.0 | 0.6 | 0.7 | 0.4 | 0.0 | 0.0 | 0.0 | 1.3 | 0.9 | 0.7 | 0.1 | 0.6 |
| 17.831 | *trans*-Carveol | 1221 | 1217^1^ | d41 | 0.0 | 0.0 | 0.0 | 0.6 | 0.0 | 0.0 | 0.0 | 0.0 | 0.0 | 0.0 | 0.0 | 0.0 |
| 17.880 | β-Cyclocitral | 1224 | 1218^1^ | d40 | 0.0 | 0.0 | 0.0 | 0.0 | 0.0 | 0.0 | 0.2 | 0.3 | 0.6 | 0.0 | 0.0 | 0.0 |
| 18.201 | Thymol methyl ether | 1237 | 1234^1^ | d2 | 0.3 | 0.0 | 0.0 | 0.0 | 0.0 | 0.0 | 0.0 | 0.0 | 0.0 | 0.0 | 0.0 | 0.0 |
| 19.250 | Carvone oxide | 1281 | 1277^2^ | b27 | 0.0 | 0.0 | 0.0 | 0.0 | 0.0 | 0.0 | 0.0 | 0.5 | 1.4 | 0.0 | 0.0 | 0.0 |
| 19.436 | Bornyl acetate | 1288 | 1284^1^ | b28 | 1.4 | 0.9 | 0.9 | 0.8 | 0.0 | 0.0 | 0.0 | 0.9 | 1.0 | 1.0 | 0.0 | 0.0 |
| 19.689 | P-Mentha-1,8-dien-7-ol | 1300 | 1296^1^ | b29 | 0.0 | 0.0 | 0.4 | 0.0 | 0.0 | 0.0 | 0.0 | 0.3 | 1.4 | 0.0 | 0.0 | 0.0 |
| 20.229 | Myrtenyl acetate | 1328 | 1329^1^ | b30 | 0.0 | 0.0 | 0.3 | 0.7 | 0.0 | 0.0 | 0.0 | 0.4 | 1.7 | 0.0 | 0.0 | 0.0 |

RT: retention time. LRI: linear retention index. LRI Lit.: LRI from literature. Codes were generated for multivariate analysis. P1: Propolis from the ‘Dois tanques’ apiary in General Carneiro municipality, P2 and P3: Propolis from the ‘Beira do mato’ apiary in General Carneiro municipality. P4 and P5: Propolis from the ‘Vila Zulmira sede’ apiary in União da Vitória municipality. P6 and P7: Propolis from the ‘Vila Zulmira serra’ apiary in União da Vitória municipality. AA: *Araucaria angustifolia* resins. PT: *Pinus taeda* resins. PE: *Pinus elliott* resins.

Table S2. Tentatively identified sesquiterpenes obtained by SHS-GCMS in brown propolis and conifer resins from southern Brazil.

| RT | Volatile | LRI | LRI Lit. | Code | Samples (% TIC) | | | | | | | | | | | |
| --- | --- | --- | --- | --- | --- | --- | --- | --- | --- | --- | --- | --- | --- | --- | --- | --- |
|  |  |  |  |  | P1 | P2 | P3 | P4 | P5 | P6 | P7 | AA1 | AA2 | PT | PE1 | PE2 |
| 20.484 | δ-Elemene | 1343 | 1340^1^ | d50 | 0.0 | 0.0 | 0.0 | 0.3 | 0.0 | 0.0 | 0.0 | 0.0 | 0.0 | 0.0 | 0.0 | 0.0 |
| 20.728 | α-Cubebene | 1355 | 1351^1^ | b31 | 1.5 | 0.4 | 1.4 | 2.8 | 0.0 | 0.0 | 0.0 | 1.6 | 0.0 | 0.0 | 0.0 | 0.0 |
| 21.149 | α-Ylangene | 1379 | 1370^1^ | d53 | 0.3 | 0.0 | 0.0 | 0.7 | 0.0 | 0.0 | 0.0 | 0.0 | 0.0 | 0.0 | 0.0 | 0.0 |
| 21.231 | α-Copaene | 1383 | 1376^1^ | d54 | 1.1 | 0.2 | 0.4 | 1.1 | 0.4 | 0.3 | 0.3 | 0.0 | 0.0 | 0.0 | 0.0 | 0.0 |
| 21.438 | β-Bourbonene | 1393 | 1384^1^ | b32 | 2.5 | 0.0 | 0.0 | 0.7 | 0.0 | 0.0 | 0.0 | 0.6 | 0.0 | 1.4 | 0.0 | 0.0 |
| 21.705 | α-Gurjunene | 1410 | 1409^1^ | d57 | 0.0 | 0.0 | 0.3 | 1.2 | 0.0 | 0.0 | 0.0 | 0.0 | 0.0 | 0.0 | 0.0 | 0.0 |
| 22.007 | *trans*-Caryophyllene | 1429 | 1420^1^ | c20 | 0.0 | 0.0 | 0.7 | 1.2 | 0.6 | 1.0 | 1.9 | 0.0 | 0.0 | 0.0 | 0.0 | 0.0 |
| 22.134 | γ-Elemene | 1438 | 1436^1^ | d61 | 0.0 | 0.0 | 0.0 | 0.6 | 0.0 | 0.0 | 0.3 | 0.0 | 0.0 | 0.0 | 0.0 | 0.0 |
| 22.163 | *trans*-α-Bergamotene | 1441 | 1435^1^ | d5 | 0.2 | 0.0 | 0.0 | 0.0 | 0.0 | 0.0 | 0.8 | 0.0 | 0.0 | 0.0 | 0.0 | 0.0 |
| 22.320 | Alloaromadendrene | 1451 | 1460^1^ | a4 | 0.0 | 0.0 | 0.0 | 0.2 | 0.0 | 0.0 | 0.0 | 0.0 | 0.0 | 0.0 | 0.0 | 0.0 |
| 22.408 | *trans*-β-Farnesene | 1456 | 1456^1^ | d64 | 0.0 | 0.0 | 0.0 | 0.3 | 0.0 | 0.0 | 0.5 | 0.0 | 0.0 | 0.0 | 0.0 | 0.0 |
| 22.476 | Cadina-3.5-diene | 1461 | 1451^3^ | d65 | 0.0 | 0.0 | 0.0 | 0.2 | 0.0 | 0.0 | 0.0 | 0.0 | 0.0 | 0.0 | 0.0 | 0.0 |
| 22.548 | α-Humulene | 1465 | 1453^1^ | c21 | 1.1 | 0.3 | 1.0 | 1.5 | 0.6 | 1.1 | 2.6 | 0.0 | 0.0 | 0.0 | 0.0 | 0.0 |
| 22.669 | α-Amorphene | 1474 | 1482^1^ | a5 | 0.0 | 0.0 | 0.0 | 0.1 | 0.0 | 0.0 | 0.0 | 0.0 | 0.0 | 0.0 | 0.0 | 0.0 |
| 22.718 | β-Acoradiene | 1477 | 1471^1^ | d66 | 0.0 | 0.0 | 0.0 | 0.0 | 0.0 | 0.0 | 0.2 | 0.0 | 0.0 | 0.0 | 0.0 | 0.0 |
| 22.834 | γ-Curcumene | 1483 | 1480^1^ | c22 | 2.2 | 0.0 | 0.0 | 1.9 | 0.3 | 0.6 | 2.8 | 0.0 | 0.0 | 0.0 | 0.0 | 0.0 |
| 23.033 | γ-Muurolene | 1486 | 1476^1^ | d68 | 0.4 | 0.0 | 0.4 | 0.7 | 0.0 | 0.0 | 0.0 | 0.0 | 0.0 | 0.0 | 0.0 | 0.0 |
| 22.951 | Germacrene D | 1492 | 1481^1^ | d74 | 0.5 | 0.0 | 0.0 | 0.7 | 0.0 | 0.0 | 0.0 | 0.0 | 0.0 | 0.0 | 0.0 | 0.0 |
| 23.041 | β-Selinene | 1497 | 1490^1^ | d72 | 0.0 | 0.0 | 0.0 | 0.0 | 0.0 | 0.5 | 2.0 | 0.0 | 0.0 | 0.0 | 0.0 | 0.0 |
| 23.156 | α-Selinene | 1506 | 1493^1^ | d76 | 0.0 | 0.0 | 0.0 | 0.0 | 0.0 | 0.0 | 2.3 | 0.0 | 0.0 | 0.0 | 0.0 | 0.0 |
| 23.179 | α-Muurolene | 1507 | 1498^1^ | c23 | 1.1 | 0.0 | 0.0 | 0.8 | 0.4 | 0.7 | 0.0 | 0.0 | 0.0 | 0.0 | 0.0 | 0.0 |
| 23.233 | β-Bisabolene | 1512 | 1508^1^ | b33 | 0.0 | 0.0 | 0.0 | 0.0 | 0.0 | 0.0 | 1.3 | 0.6 | 2.6 | 0.0 | 0.0 | 0.0 |
| 23.273 | β-Curcumene | 1515 | 1513^1^ | d78 | 0.0 | 0.0 | 0.0 | 0.0 | 0.0 | 0.0 | 2.3 | 0.0 | 0.0 | 0.0 | 0.0 | 0.0 |
| 23.360 | *cis*-γ-Bisabolene | 1521 | 1512^1^ | d79 | 0.0 | 0.0 | 0.0 | 0.0 | 0.0 | 0.0 | 0.5 | 0.0 | 0.0 | 0.0 | 0.0 | 0.0 |
| 23.405 | γ-Cadinene | 1525 | 1513^1^ | d55 | 1.2 | 0.0 | 0.0 | 0.7 | 0.0 | 0.0 | 0.0 | 0.0 | 0.0 | 0.0 | 0.0 | 0.0 |
| 23.508 | δ-Cadinene | 1531 | 1523^1^ | b34 | 2.4 | 0.4 | 1.1 | 2.9 | 0.6 | 0.8 | 1.1 | 1.4 | 0.7 | 0.0 | 0.0 | 0.0 |
| 23.655 | 1.4-Cadinadiene | 1543 | 1529^4^ | b35 | 0.3 | 0.0 | 0.0 | 0.9 | 0.0 | 0.0 | 0.0 | 0.4 | 0.7 | 0.0 | 0.0 | 0.0 |
| 23.692 | *trans*-α-Bisabolene | 1546 | 1540^1^ | d81 | 0.0 | 0.0 | 0.0 | 0.0 | 0.0 | 0.0 | 2.1 | 0.0 | 0.0 | 0.0 | 0.0 | 0.0 |
| 23.818 | α-Calacorene | 1555 | 1545^1^ | d83 | 0.0 | 0.0 | 0.0 | 0.4 | 0.0 | 0.6 | 0.6 | 0.0 | 0.0 | 0.0 | 0.0 | 0.0 |
| 24.317 | Salvial-4(14)-en-1-one | 1592 | 1593^1^ | d85 | 0.6 | 0.0 | 0.0 | 0.9 | 0.0 | 1.3 | 0.0 | 0.0 | 0.0 | 0.0 | 0.0 | 0.0 |
| 24.607 | Lepidozene | 1615 | 1628^5^ | d75 | 0.0 | 0.0 | 0.0 | 0.0 | 0.0 | 0.3 | 0.6 | 0.0 | 0.0 | 0.0 | 0.0 | 0.0 |
| 24.808 | Cadina-1(10).6.8-triene | 1632 | 1625^6^ | d88 | 0.0 | 0.0 | 0.0 | 0.0 | 0.0 | 0.0 | 0.4 | 0.0 | 0.0 | 0.0 | 0.0 | 0.0 |
| 24.945 | Epicubenol | 1643 | 1638^7^ | d89 | 0.0 | 0.0 | 0.0 | 0.6 | 0.0 | 0.0 | 0.0 | 0.0 | 0.0 | 0.0 | 0.0 | 0.0 |
| 24.994 | γ-Eudesmol | 1646 | 1632^1^ | d90 | 0.0 | 0.0 | 0.0 | 0.0 | 2.5 | 5.7 | 6.1 | 0.0 | 0.0 | 0.0 | 0.0 | 0.0 |
| 25.096 | Hinesol | 1655 | 1640^8^ | d91 | 0.3 | 0.0 | 0.0 | 0.0 | 0.2 | 0.7 | 1.0 | 0.0 | 0.0 | 0.0 | 0.0 | 0.0 |
| 25.263 | β-Eudesmol | 1669 | 1652^1^ | b36 | 0.3 | 0.0 | 0.3 | 0.7 | 3.3 | 6.7 | 8.1 | 0.7 | 2.4 | 0.0 | 0.0 | 0.0 |
| 25.542 | α-Bisabolol | 1691 | 1683^1^ | b37 | 0.7 | 0.4 | 1.3 | 1.6 | 2.2 | 2.9 | 9.7 | 4.1 | 12.8 | 0.0 | 0.0 | 0.0 |

RT: retention time. LRI: linear retention index. LRI Lit.: LRI from literature. Codes were generated for multivariate analysis. P1: Propolis from the ‘Dois tanques’ apiary in General Carneiro municipality, P2 and P3: Propolis from the ‘Beira do mato’ apiary in General Carneiro municipality. P4 and P5: Propolis from the ‘Vila Zulmira sede’ apiary in União da Vitória municipality. P6 and P7: Propolis from the ‘Vila Zulmira serra’ apiary in União da Vitória municipality. AA: *Araucaria angustifolia* resins. PT: *Pinus taeda* resins. PE: *Pinus elliott* resins.

Table S3. Tentatively identified aldehydes, hydrocarbons, alcohols, ketones, esters, acids and ethers obtained by SHS-GCMS in brown propolis and conifer resins from southern Brazil.

| RT | Volatile | LRI | LRI Lit. | Code | Samples (% TIC) | | | | | | | | | | | |
| --- | --- | --- | --- | --- | --- | --- | --- | --- | --- | --- | --- | --- | --- | --- | --- | --- |
|  |  |  |  |  | P1 | P2 | P3 | P4 | P5 | P6 | P7 | AA1 | AA2 | PT | PE1 | PE2 |
| 6.006 | Furfural | 875 | 868^2^ | c6 | 0.5 | 0.0 | 0.8 | 0.4 | 4.9 | 0.4 | 0.0 | 0.0 | 0.0 | 0.0 | 0.0 | 0.0 |
| 7.671 | Styrene | 896 | 891^1^ | d14 | 0.0 | 0.0 | 0.0 | 0.0 | 0.8 | 0.8 | 0.5 | 0.0 | 0.0 | 0.0 | 0.0 | 0.0 |
| 9.815 | Benzaldehyde | 958 | 963^1^ | c7 | 0.0 | 0.0 | 0.0 | 0.5 | 3.0 | 1.9 | 0.8 | 0.0 | 0.0 | 0.0 | 0.0 | 0.0 |
| 9.903 | 5-Methylfurfural | 961 | 967^1^ | c8 | 0.0 | 0.0 | 0.0 | 0.3 | 1.6 | 0.0 | 0.0 | 0.0 | 0.0 | 0.0 | 0.0 | 0.0 |
| 12.156 | Benzyl alcohol | 1032 | 1037^1^ | c10 | 0.0 | 0.0 | 0.0 | 0.0 | 0.3 | 1.6 | 1.2 | 0.0 | 0.0 | 0.0 | 0.0 | 0.0 |
| 13.199 | Acetophenone | 1065 | 1067^1^ | c12 | 0.0 | 0.0 | 0.0 | 0.0 | 0.3 | 0.6 | 0.3 | 0.0 | 0.0 | 0.0 | 0.0 | 0.0 |
| 14.356 | Nonanal | 1102 | 1103^1^ | d17 | 0.3 | 0.0 | 0.0 | 0.1 | 0.0 | 0.0 | 0.0 | 0.0 | 0.0 | 0.0 | 0.0 | 0.0 |
| 14.583 | 1-octen-3-yl acetate | 1110 | 1110^1^ | a3 | 0.0 | 0.0 | 0.0 | 0.4 | 0.0 | 0.0 | 0.0 | 0.0 | 2.7 | 0.0 | 0.0 | 0.0 |
| 14.651 | Benzeneethanol | 1111 | 1115^1^ | c14 | 0.0 | 0.0 | 0.0 | 0.0 | 0.0 | 2.1 | 1.5 | 0.0 | 0.0 | 0.0 | 0.0 | 0.0 |
| 16.522 | Octanoic acid | 1173 | 1182^1^ | d32 | 0.0 | 0.0 | 0.0 | 0.0 | 0.0 | 0.4 | 0.2 | 0.0 | 0.0 | 0.0 | 0.0 | 0.0 |
| 17.402 | Decanal | 1203 | 1205^1^ | d38 | 0.3 | 0.0 | 0.0 | 0.0 | 0.0 | 0.0 | 0.0 | 0.0 | 0.0 | 0.0 | 0.0 | 0.0 |
| 17.765 | Coumaran | 1218 | 1226^9^ | b25 | 0.0 | 0.0 | 0.0 | 0.0 | 0.7 | 2.5 | 1.3 | 0.0 | 0.0 | 0.0 | 0.0 | 0.0 |
| 17.914 | Cumin aldehyde | 1225 | 1235^1^ | ta2 | 0.0 | 0.0 | 0.0 | 0.0 | 0.0 | 0.0 | 0.0 | 0.0 | 0.0 | 2.0 | 0.8 | 0.8 |
| 19.795 | *trans*-Cinnamyl alcohol | 1305 | 1311^10^ | d49 | 0.0 | 0.0 | 0.0 | 0.0 | 0.0 | 2.0 | 2.7 | 0.0 | 0.0 | 0.0 | 0.0 | 0.0 |
| 19.971 | 4-Vinylguaiacol | 1314 | 1317^1^ | c17 | 0.0 | 0.0 | 0.0 | 0.0 | 0.8 | 2.2 | 0.8 | 0.0 | 0.0 | 0.0 | 0.0 | 0.0 |
| 20.800 | Eugenol | 1360 | 1358^1^ | d51 | 0.0 | 0.0 | 0.0 | 0.0 | 0.0 | 0.0 | 0.5 | 0.0 | 0.0 | 0.0 | 0.0 | 0.0 |
| 20.858 | Decanoic acid | 1363 | 1369^1^ | d52 | 0.0 | 0.0 | 0.0 | 0.0 | 0.0 | 0.5 | 0.3 | 0.0 | 0.0 | 0.0 | 0.0 | 0.0 |
| 21.390 | Ethyl decanoate | 1392 | 1395^1^ | d56 | 0.0 | 0.0 | 0.0 | 0.0 | 0.0 | 0.0 | 0.2 | 0.0 | 0.0 | 0.0 | 0.0 | 0.0 |
| 21.543 | Vanillin | 1400 | 1405^1^ | c19 | 2.5 | 0.8 | 0.8 | 0.7 | 0.5 | 0.0 | 0.0 | 0.0 | 0.0 | 0.0 | 0.0 | 0.0 |
| 21.922 | *trans*-Cinnamic acid | 1424 | 1433^2^ | d58 | 0.0 | 0.0 | 0.0 | 0.3 | 0.3 | 0.5 | 0.0 | 0.0 | 0.0 | 0.0 | 0.0 | 0.0 |
| 22.236 | *trans*-Cinnamyl acetate | 1446 | 1446^10^ | d62 | 0.0 | 0.0 | 0.0 | 0.0 | 0.0 | 0.0 | 0.3 | 0.0 | 0.0 | 0.0 | 0.0 | 0.0 |
| 22.937 | Acetovanillone | 1491 | 1490^11^ | d70 | 0.0 | 0.0 | 0.0 | 0.0 | 0.0 | 0.0 | 0.4 | 0.0 | 0.0 | 0.0 | 0.0 | 0.0 |
| 26.517 | Benzyl benzoate | 1778 | 1761^1^ | d97 | 0.0 | 0.0 | 0.0 | 0.8 | 1.7 | 0.0 | 0.0 | 0.0 | 0.0 | 0.0 | 0.0 | 0.0 |

RT: retention time. LRI: linear retention index. LRI Lit.: LRI from literature. Codes were generated for multivariate analysis. P1: Propolis from the ‘Dois tanques’ apiary in General Carneiro municipality, P2 and P3: Propolis from the ‘Beira do mato’ apiary in General Carneiro municipality. P4 and P5: Propolis from the ‘Vila Zulmira sede’ apiary in União da Vitória municipality. P6 and P7: Propolis from the ‘Vila Zulmira serra’ apiary in União da Vitória municipality. AA: *Araucaria angustifolia* resins. PT: *Pinus taeda* resins. PE: *Pinus elliott* resins.

Table S4. Not identified volatiles and unidentified terpene obtained by SHS-GCMS in brown propolis and conifer resins from southern Brazil.

| RT | Volatile | LRI | Code | Samples (% TIC) | | | | | | | | | | | |
| --- | --- | --- | --- | --- | --- | --- | --- | --- | --- | --- | --- | --- | --- | --- | --- |
|  |  |  |  | P1 | P2 | P3 | P4 | P5 | P6 | P7 | AA1 | AA2 | PT | PE1 | PE2 |
| 2.233 | Not identified | - | ta1 | 0.0 | 0.0 | 0.0 | 0.8 | 1.7 | 1.0 | 0.9 | 0.0 | 0.0 | 1.3 | 0.8 | 1.0 |
| 2.294 | Not identified | - | a1 | 0.0 | 0.0 | 2.0 | 0.0 | 0.0 | 0.0 | 0.0 | 0.0 | 0.0 | 0.0 | 0.0 | 0.0 |
| 2.460 | Not identified | - | d18 | 8.8 | 5.2 | 0.0 | 0.0 | 0.0 | 0.0 | 0.0 | 0.0 | 0.0 | 0.0 | 0.0 | 0.0 |
| 2.421 | Not identified | - | c2 | 0.0 | 0.0 | 0.0 | 0.5 | 2.6 | 1.7 | 0.7 | 0.0 | 0.0 | 0.0 | 0.0 | 0.0 |
| 2.630 | Not identified | - | c3 | 0.0 | 0.0 | 0.0 | 0.0 | 0.0 | 0.0 | 0.6 | 0.0 | 0.0 | 0.0 | 0.0 | 0.0 |
| 2.761 | Not identified | - | c13 | 0.0 | 0.0 | 0.0 | 0.0 | 0.5 | 0.0 | 0.0 | 0.0 | 0.0 | 0.0 | 0.0 | 0.0 |
| 2.848 | Not identified | - | c4 | 0.0 | 0.0 | 0.0 | 0.0 | 0.6 | 0.3 | 0.3 | 0.0 | 0.0 | 0.0 | 0.0 | 0.0 |
| 3.210 | Not identified | - | d4 | 0.0 | 0.0 | 0.0 | 0.0 | 0.6 | 0.0 | 0.0 | 0.0 | 0.0 | 0.0 | 0.0 | 0.0 |
| 3.635 | Not identified | - | d8 | 0.0 | 0.0 | 0.0 | 0.0 | 0.0 | 0.4 | 0.4 | 0.0 | 0.0 | 0.0 | 0.0 | 0.0 |
| 3.750 | Not identified | - | d9 | 0.0 | 0.0 | 0.0 | 0.0 | 0.6 | 3.6 | 2.2 | 0.0 | 0.0 | 0.0 | 0.0 | 0.0 |
| 4.447 | Not identified | - | d10 | 0.0 | 0.0 | 0.0 | 0.0 | 0.0 | 0.0 | 0.3 | 0.0 | 0.0 | 0.0 | 0.0 | 0.0 |
| 4.475 | Not identified | - | d11 | 0.0 | 0.4 | 0.3 | 0.0 | 0.0 | 0.0 | 0.0 | 0.0 | 0.0 | 0.0 | 0.0 | 0.0 |
| 4.570 | Not identified | - | d12 | 0.0 | 0.0 | 0.0 | 0.0 | 0.6 | 3.8 | 2.3 | 0.0 | 0.0 | 0.0 | 0.0 | 0.0 |
| 5.150 | Not identified | - | c5 | 0.4 | 0.0 | 0.0 | 0.0 | 0.7 | 0.5 | 0.2 | 0.0 | 0.0 | 0.0 | 0.0 | 0.0 |
| 6.116 | Not identified | 877 | d13 | 0.0 | 0.5 | 0.3 | 0.0 | 0.0 | 0.0 | 0.0 | 0.0 | 0.0 | 0.0 | 0.0 | 0.0 |
| 10.859 | Not identified | 991 | c9 | 0.0 | 0.8 | 0.0 | 0.0 | 0.6 | 0.9 | 0.0 | 0.0 | 0.0 | 0.0 | 0.0 | 0.0 |
| 15.598 | Not identified | 1143 | d22 | 0.0 | 0.0 | 0.0 | 0.0 | 0.4 | 0.5 | 0.5 | 0.0 | 0.0 | 0.0 | 0.0 | 0.0 |
| 15.846 | Not identified | 1151 | d26 | 0.0 | 0.0 | 0.0 | 0.0 | 0.4 | 0.7 | 0.5 | 0.0 | 0.0 | 0.0 | 0.0 | 0.0 |
| 16.370 | Not identified | 1168 | c15 | 0.0 | 0.0 | 0.0 | 0.0 | 5.7 | 3.8 | 2.2 | 0.0 | 0.0 | 0.0 | 0.0 | 0.0 |
| 16.875 | Not identified | 1185 | d36 | 0.0 | 0.0 | 0.4 | 0.0 | 0.0 | 0.0 | 0.0 | 0.0 | 0.0 | 0.0 | 0.0 | 0.0 |
| 17.409 | Not identified | 1205 | d44 | 0.0 | 0.0 | 0.3 | 0.0 | 0.0 | 0.0 | 0.0 | 0.0 | 0.0 | 0.0 | 0.0 | 0.0 |
| 17.706 | Not identified | 1216 | d39 | 0.0 | 0.0 | 0.0 | 0.0 | 0.0 | 0.5 | 0.7 | 0.0 | 0.0 | 0.0 | 0.0 | 0.0 |
| 17.847 | Not identified | 1222 | b17 | 0.0 | 0.7 | 0.7 | 0.0 | 0.0 | 0.0 | 0.0 | 0.0 | 0.0 | 0.0 | 0.0 | 0.0 |
| 19.327 | Not identified | 1284 | d3 | 0.0 | 0.0 | 0.0 | 0.0 | 0.0 | 0.0 | 0.4 | 0.0 | 0.0 | 0.0 | 0.0 | 0.0 |
| 19.466 | Unidentified terpene | 1290 | c16 | 0.0 | 0.0 | 0.0 | 0.0 | 0.4 | 1.4 | 2.1 | 0.0 | 0.0 | 0.0 | 0.0 | 0.0 |
| 19.678 | Not identified | 1299 | d47 | 0.0 | 0.0 | 0.0 | 0.6 | 0.0 | 0.0 | 0.0 | 0.0 | 0.0 | 0.0 | 0.0 | 0.0 |
| 22.140 | Not identified | 1439 | d59 | 0.7 | 0.0 | 0.0 | 0.0 | 0.0 | 0.0 | 0.0 | 0.0 | 0.0 | 0.0 | 0.0 | 0.0 |
| 23.768 | Not identified | 1552 | d82 | 0.0 | 0.0 | 0.0 | 0.0 | 0.0 | 0.7 | 0.0 | 0.0 | 0.0 | 0.0 | 0.0 | 0.0 |
| 24.255 | Not identified | 1588 | d84 | 0.6 | 0.0 | 0.0 | 0.0 | 0.0 | 0.0 | 0.0 | 0.0 | 0.0 | 0.0 | 0.0 | 0.0 |
| 24.366 | Not identified | 1596 | d86 | 0.4 | 0.0 | 0.0 | 0.5 | 0.0 | 0.0 | 0.0 | 0.0 | 0.0 | 0.0 | 0.0 | 0.0 |
| 24.755 | Not identified | 1628 | d87 | 0.0 | 0.0 | 0.0 | 0.4 | 0.0 | 0.0 | 0.0 | 0.0 | 0.0 | 0.0 | 0.0 | 0.0 |
| 25.481 | Not identified | 1687 | d95 | 0.0 | 0.0 | 0.0 | 0.0 | 0.0 | 0.5 | 0.0 | 0.0 | 0.0 | 0.0 | 0.0 | 0.0 |
| 25.679 | Not identified | 1703 | d96 | 0.0 | 0.0 | 0.0 | 0.3 | 0.0 | 0.0 | 0.2 | 0.0 | 0.0 | 0.0 | 0.0 | 0.0 |
| 26.161 | Not identified | 1746 | d15 | 0.4 | 0.0 | 0.0 | 0.0 | 0.0 | 0.0 | 0.0 | 0.0 | 0.0 | 0.0 | 0.0 | 0.0 |
| 27.148 | Not identified | 1836 | d98 | 0.0 | 0.0 | 0.0 | 0.0 | 0.7 | 1.0 | 1.3 | 0.0 | 0.0 | 0.0 | 0.0 | 0.0 |
| 27.814 | Not identified | 1894 | d80 | 0.0 | 0.0 | 0.5 | 0.2 | 0.7 | 0.6 | 1.3 | 0.0 | 0.0 | 0.0 | 0.0 | 0.0 |
| 27.901 | Not identified | 1904 | b38 | 0.0 | 0.0 | 0.0 | 0.5 | 2.0 | 1.4 | 0.0 | 0.8 | 0.0 | 0.0 | 0.0 | 0.0 |
| 28.436 | Not identified | 1928 | b39 | 0.0 | 0.0 | 0.5 | 0.5 | 1.9 | 1.0 | 0.5 | 0.7 | 0.0 | 0.0 | 0.0 | 0.0 |
| 28.579 | Not identified | 1938 | d100 | 0.0 | 0.0 | 0.0 | 0.4 | 0.8 | 1.7 | 0.3 | 0.0 | 0.0 | 0.0 | 0.0 | 0.0 |
| 28.957 | Not identified | 1958 | ta3 | 0.0 | 0.0 | 0.0 | 0.0 | 0.0 | 0.0 | 0.0 | 0.0 | 0.0 | 0.6 | 0.0 | 0.0 |
| 29.706 | Not identified | 1996 | d101 | 0.3 | 0.0 | 0.0 | 0.0 | 0.8 | 0.8 | 0.6 | 0.0 | 0.0 | 0.0 | 0.0 | 0.0 |
| 29.866 | Not identified | - | d102 | 0.0 | 0.0 | 0.0 | 0.0 | 0.3 | 0.3 | 0.0 | 0.0 | 0.0 | 0.0 | 0.0 | 0.0 |

RT: retention time. LRI: linear retention index. - : out of range to calculate the LRI according to Babushok et al. (2011) (LRI = (([RT – T_n_]/[T_n+1_ – T_n_]) + n) * 100), with RT as the retention time of the compound, T_n_ as the retention time of the alkane eluting before, T _n+1_ as the retention time of alkane eluting after and n as the number of carbon atoms in previous alkane). Codes were generated for multivariate analysis. PT: *Pinus taeda*. PE: *Pinus elliott*; AA: *Araucaria angustifolia*. P1, P2 and P3: propolis samples from General Carneiro municipality. P4 and P5: propolis samples from ‘Vila Zulmira sede’ apiary. P6 and P7: propolis samples from ‘Vila Zulmira serra’ apiary.

**References**

1. Babushok, V. I., Linstrom, P. J. & Zenkevich, I. G. Retention Indices for Frequently Reported Compounds of Plant Essential Oils. *J. Phys. Chem. Ref. Data* **40**, (2011).

2. Mottram, R. LRI & Odour Database. http://www.odour.org.uk/index.html.

3. Matejić, J. S., Džamić, A. M., Ristić, M. S., Ranđelović, V. N. & Marin, P. D. Essential Oil Composition of Cachrys cristata – a rare and Endangered Species in the Flora of Serbia. *Nat. Prod. Commun.* **7**, 235–236 (2012).

4. Lei, H. *et al.* Composition and variability of essential oils of Platycladus orientalis growing in China. *Biochem. Syst. Ecol.* **38**, 1000–1006 (2010).

5. Mohadjerani, M., Hosseinzadeh, R. & Hosseini, M. Chemical composition and antibacterial properties of essential oil and fatty acids of different parts of Ligularia persica Boiss. *Avicenna J. Phytomedicine* **6**, 357–365 (2016).

6. Ramírez-Rueda, R. Y., Marinho, J. & Salvador, M. J. Bioguided identification of antimicrobial compounds from Chrysopogon zizaniodes (L.) Roberty root essential oil. *Future Microbiol.* **14**, 1179–1189 (2019).

7. Shellie, R., Mondello, L., Marriott, P. & Dugo, G. Characterisation of lavender essential oils by using gas chromatography-mass spectrometry with correlation of linear retention indices and comparison with comprehensive two-dimensional gas chromatography. *J. Chromatogr. A* **970**, 225–234 (2002).

8. Andrade, E. H. A., Alves, C. N., Guimarães, E. F., Carreira, L. M. M. & Maia, J. G. S. Variability in essential oil composition of Piper dilatatum L.C. Rich. *Biochem. Syst. Ecol.* **39**, 669–675 (2011).

9. Nafis, A. *et al.* Antioxidant activity and evidence for synergism of Cannabis sativa (L.)essential oil with antimicrobial standards. *Ind. Crops Prod.* **137**, 396–400 (2019).

10. Kim, J. *et al.* Fumigant and contact toxicity of 22 wooden essential oils and their major components against Drosophila suzukii (Diptera: Drosophilidae). *Pestic. Biochem. Physiol.* **133**, 35–43 (2016).

11. Vichi, S. *et al.* Volatile and semi-volatile components of oak wood chips analysed by Accelerated Solvent Extraction (ASE) coupled to gas chromatography-mass spectrometry (GC-MS). *Food Chem.* **102**, 1260–1269 (2007).
